# Soil bacterial community composition in rice–fish integrated farming systems with different planting years

**DOI:** 10.1038/s41598-021-90370-9

**Published:** 2021-05-25

**Authors:** Zheng Zhao, Changbin Chu, Deping Zhou, Qingfeng Wang, Shuhang Wu, Xianqing Zheng, Ke Song, Weiguang Lv

**Affiliations:** Eco-Environmental Protection Institute of Shanghai Academy of Agricultural Sciences, 1000 Jinqi Rd., Shanghai, 201403 People’s Republic of China

**Keywords:** Agroecology, Microbial ecology

## Abstract

The high productivity and efficient nutrient utilization in rice–fish integrated farming system are well reported. However, the characteristics of soil bacterial communities and their relationship with soil nutrient availability in rice–fish field remain unclear. In this study, we selected three paddy fields, including a rice monoculture field and two rice–fish fields with different planting years, to investigate the soil bacterial community composition with Illumina MiSeq sequencing technology. The results indicated that the soil properties were significantly different among different rice farming systems. The soil bacterial community composition in the rice–fish field was significantly different from that in the rice monoculture field. Five of the top 15 phyla were observed with significant differences and *Nitrospirae* was the most significant one. However, no taxa observed with significance between the rice planting area and aquaculture area no matter in the 1st or 5th year of rice–fish field. RDA analysis showed that the soil bacterial community differentiation in the 5th year of rice–fish field was positively correlated with soil properties, such as AN and OM contents, EC and pH value. Although the rice yields in rice–fish field decreased, the net economic benefit of the rice–fish system enhanced obviously due to the high value of aquaculture animals.

## Introduction

The rice–fish integrated farming system is an ancient agricultural production pattern and has been practiced for more than 2000 years in China^1, 2^. In 2005, this system was recognized as a globally important agricultural heritage system (GIAHS)^3^. Recently, the use of rice–fish integrated farming has been gradually rising due to its exceptionally benefits, including positive effects on weed and pest control, reduced agrochemical inputs, improved soil nutrient availability, and increased yields and economic benefits^4–6^. The area of rice–fish fields in China is 1.67 × 10^6^ ha, which accounts for approximately 4.5% of the total rice planting area in China^7^. Many studies have demonstrated that rice–fish integrated farming is an economical and eco-friendly agricultural pattern alternative to rice monoculture^8, 9^.

Microorganisms play crucial roles in the formation of soil structure, decomposition of soil organic matter, circulation of soil nutrients, and crop health and growth^10^. The soil microbial community composition can reflect the quality of the soil ecological environment and directly affect soil fertility^11^. However, most previous studies related to rice–fish integrated farming systems have focused only on productivity or environmental benefits^12, 13^ rather than complex soil organisms such as soil microbial communities. To date, there are few reports available about the characterization of the soil bacterial diversity and composition in rice–fish integrated farming systems. Therefore, it is necessary to study the community characteristics of soil microorganisms in rice–fish system, as well as their relationship with soil properties and productivity.

Chongming Eco-island, the third largest island in China, is located in the northeast of Shanghai city. Rice–fish integrated farming was introduced to the island in 2005, and yellow finless eel (*Monopterus albus*) and loach (*Misgurnus* spp.) are the main fish species cultured in paddy fields. In this study, the evolutionary characteristics of the soil bacterial communities in the rice–fish integrated farming systems were investigated using Illumina MiSeq sequencing. Our hypothesis is the soil bacterial community composition in rice–fish integrated farming system was greatly changed compared with rice monoculture. These differentiations of soil bacterial may relate to some soil factors or crucial taxa, which may further influence the productivity of the system. To test this hypothesis, a rice monoculture field and 2 rice–fish fields with different planting years (1 year and 5 years, respectively) on Chongming Eco-island were choose to investigate the soil bacterial community composition.

## Results

### Soil properties in different rice farming systems

Five treatments were designed in the three selected rice fields, including (1) rice monoculture field (RM); (2) planting area in the 1st year of rice–fish field (OP); (3) aquaculture area in the 1st year of rice–fish field (OA); (4) planting area in the 5th year of rice–fish field (FP); (5) aquaculture area in the 5th year of rice–fish field (FA). The soil properties of the five treatments were shown in Table 1. The highest soil available nitrogen (AN) content was observed in FP and was significantly higher than that in RM, OP and OA. The highest soil available phosphorus (AP) content was observed in RM and was significantly higher than that in the other 4 treatments. The highest soil available potassium (AK) content was measured in the 1st year of rice–fish field (OP and OA), followed by RM and the 5th year of rice–fish field (FP and FA), and significant differences were observed among different rice fields. The highest soil organic matter (OM) content appeared in the 5th year of rice–fish field (FP and FA), and was only significantly higher than that in OA. In addition, the soil pH in the 1st year of rice–fish field (OP and OA) was significantly lower than that in RM and the 5th year of rice–fish field (FP and FA). In summary, significant differences of soil properties were observed among the different rice farming systems.

**Table 1:** Soil properties in different rice systems and areas.

Treatments	AN (mg/kg)	AP (mg/kg)	AK (mg/kg)	OM (%)	pH
RM	193.67 ± 12.12 c	13.44 ± 0.84 a	70.60 ± 3.72 b	1.21 ± 0.15 ab	7.99 ± 0.10 a
OP	197.65 ± 8.48 c	5.95 ± 1.44 b	150.40 ± 9.56 a	1.02 ± 0.10 ab	7.27 ± 0.18 b
OA	209.59 ± 9.55 bc	6.28 ± 1.66 b	146.40 ± 16.44 a	0.98 ± 0.12 b	7.25 ± 0.10 b
FP	246.73 ± 18.51 a	4.77 ± 1.11 b	29.60 ± 1.62 c	1.24 ± 0.11 a	8.00 ± 0.05 a
FA	225.51 ± 10.04 ab	5.59 ± 0.94 b	23.40 ± 3.93 c	1.24 ± 0.07 a	7.83 ± 0.16 a

Different letters within columns indicate significant differences at *P* < 0.05.

### Soil bacterial community composition

**Figure 1:**
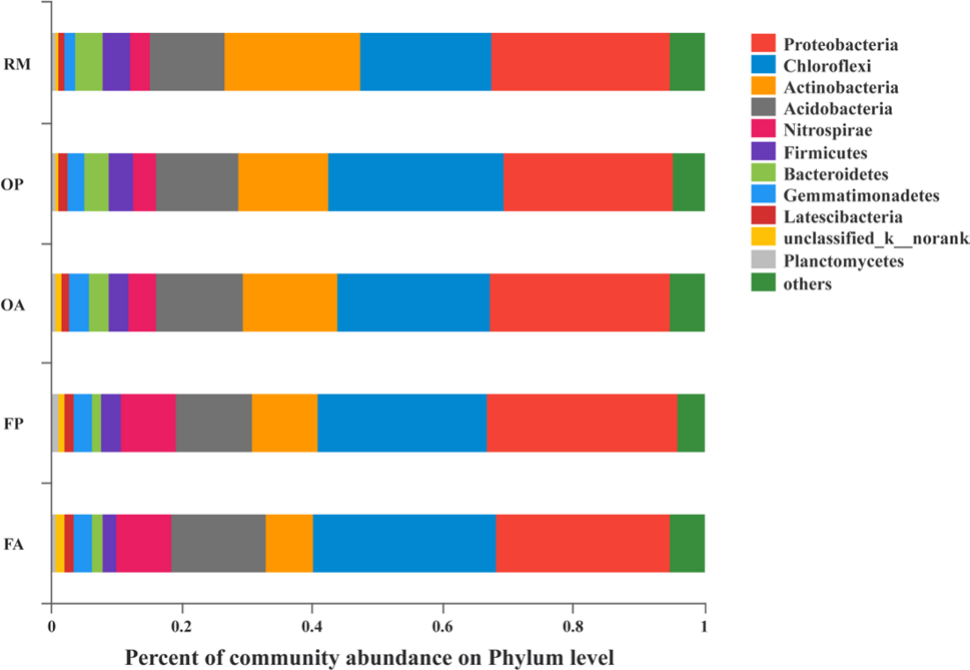
The average relative abundances on phylum level of soil bacterial communities in different rice systems and areas.

**Figure 2:**
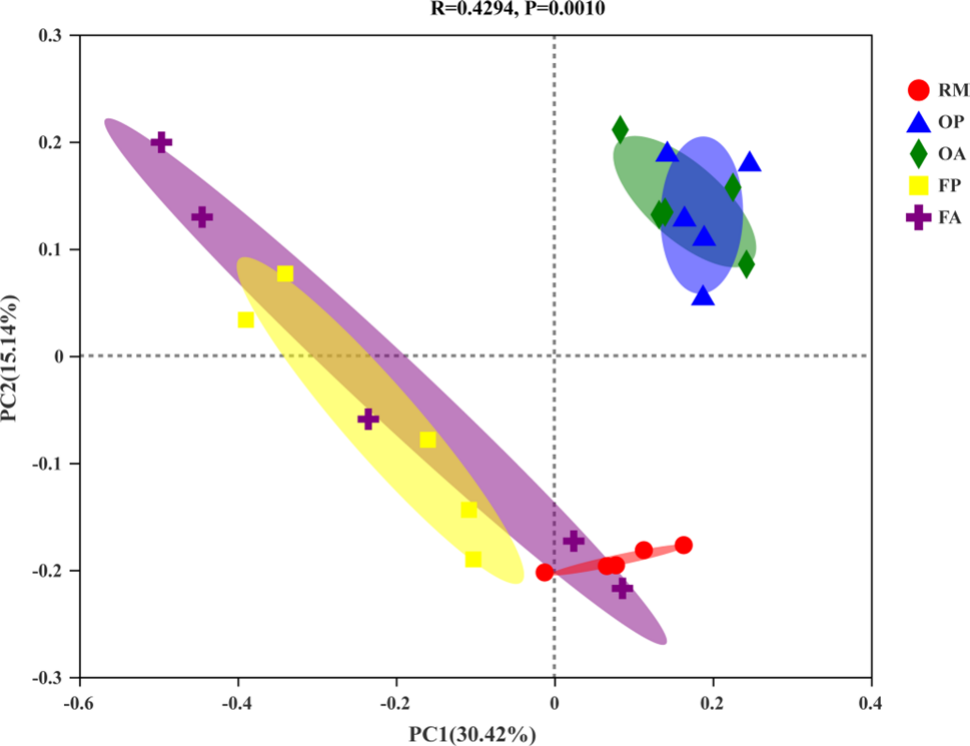
PCoA analysis on OTU level based on bray_curtis distance algorithm (significance among treatments were conducted with ANOSIM test, R = 0.4294, *P* = 0.0010).

A total of 1,346,468 sequences were obtained by 16S rRNA MiSeq sequencing analysis after basal quality control (reads containing ambiguous bases were discarded; only overlapping sequences longer than 10 bp were assembled; Operational taxonomic units (OTUs) were clustered with 97% similarity). These sequences were classified as 46 phyla, 800 genera and 5335 OTUs. As shown in Fig. 1, the dominant bacterial phyla across different treatments were *Proteobacteria* (26.06–29.41%) and *Chloroflexi* (20.07–27.99%), followed by *Actinobacteria* (7.22–20.87%), *Acidobacteria* (11.36–14.46%) and *Nitrospirae* (3.11–8.50%). Since the implementation of rice–fish farming regime, the soil bacterial community composition has greatly changed. For example, *Actinobacteria* abundance decreased from 20.87% in RM to 7.22% in FA, while *Nitrospirae* abundance greatly increased from 3.11% in RM to 8.50% in FA. Between different areas in a same rice–fish field (i.e. OP vs OA or FP vs FA), the bacterial community composition were similar. The PCoA analysis on OTU level also showed that different areas within the same rice–fish field had high similarity in bacterial community composition. In contrast, the bacterial community composition differed distinctly among different rice farming systems (Fig. 2). Bacterial alpha diversity indices, as evaluated by Shannon, Simpson, ACE and Chao1, were shown in Table 2. Student’s t-test was adopted to evaluate the difference among treatments. The results showed that the alpha indices of FP were significantly lower than other treatments, except for Simpson index.

**Table 2:** Alpha diversity indices of soil bacterial in different rice systems and areas.

Treatments	Shannon	Simpson	ACE	Chao1
RM	6.73 ab	0.0066 a	3451.68 ab	3476.27 ab
OP	6.97 a	0.0021 a	3450.48 ab	3488.81 ab
OA	6.93 a	0.0023 a	3444.52 a	3486.72 a
FP	6.59 b	0.0044 a	3115.12 b	3153.36 b
FA	6.60 ab	0.0049 a	3100.57 ab	3098.53 ab

Different letters within columns indicate significant differences at *P* < 0.05.

**Figure 3:**
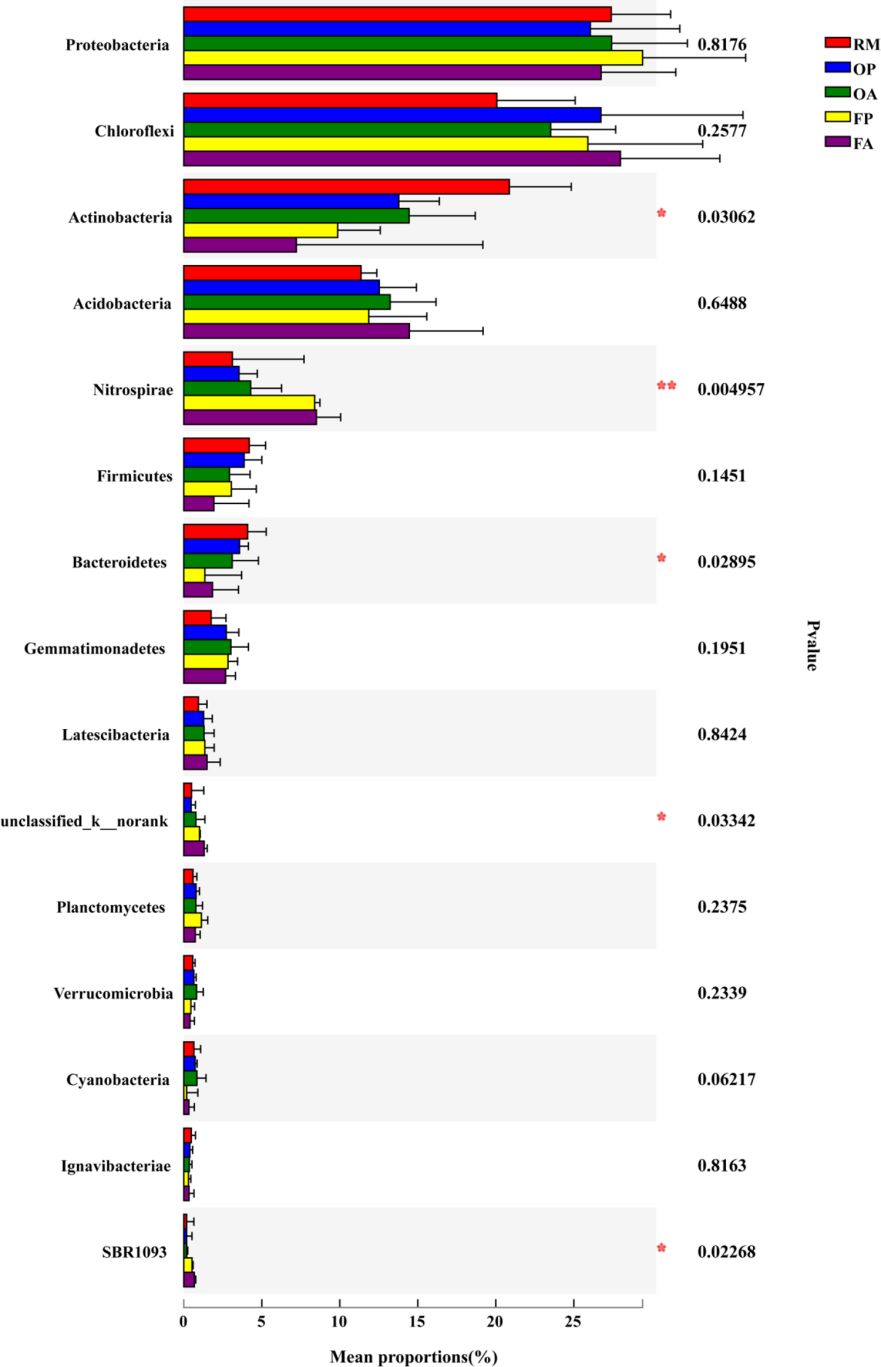
The differences with significance of the top 15 phyla in different rice systems and areas (* indicates 0.01 < *P* ≤ 0.05, ** indicates 0.001 < *P* ≤ 0.01).

**Figure 4:**
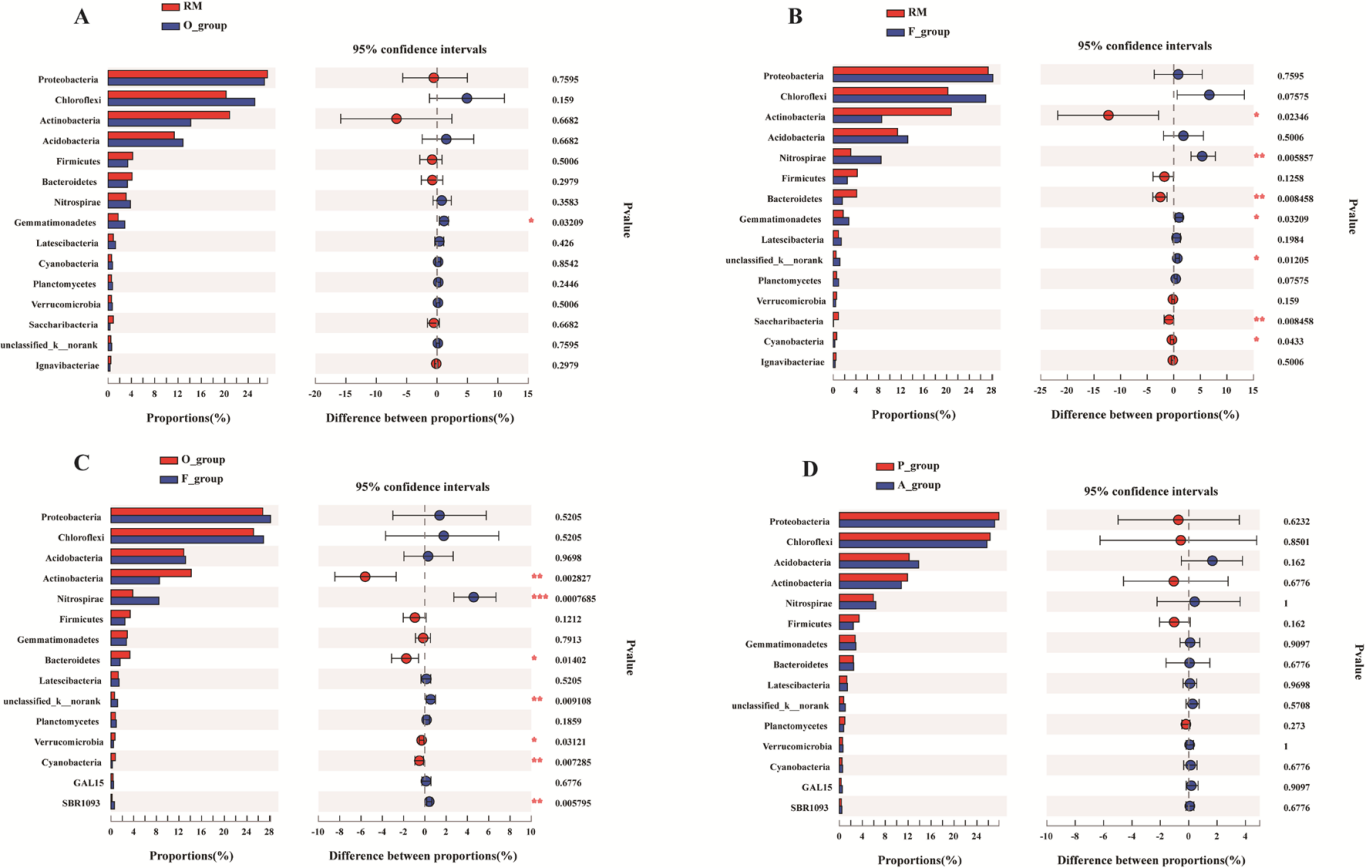
The differences with significance of the top 15 phyla between pairs of experimental groups (A: RM vs O_group, B: RM vs F_group, C: O_group vs F_group, D: P_group vs A_group; * indicates 0.01 < *P* ≤ 0.05, ** indicates 0.001 < *P* ≤ 0.01, *** *P* ≤ 0.001).

Based on the Kruskal–Wallis test, the statistical differences among treatments were evaluated in the abundances of the top 15 phyla. The results showed that 5 phyla, including *Actinobacteria*, *Nitrospirae*, *Bacteroidetes*, *Unclassified_k_norank* and *SBR1093* were observed significant differences among treatments, and the most significant phylum was *Nitrospirae* (Fig. 3). In order to trace the source of the significant differences, the Wilcoxon tests were conducted between every two rice cultivation patterns separately (Fig. 4). The results indicated that the significant differences were mainly derived from the comparison between RM and F_group (FP & FA), as well as the comparison between the O_group (OP & OA) and F_group. In the comparison between the RM and O_group, only the phylum *Gemmatimonadetes* was observed to have a significant difference. Furthermore, we also compared the differences of the top 15 phyla between planting area (P_group) and aquaculture area (A_group) within rice–fish fields, and the results showed no phyla observed with significant differences in the abundances.

### Cluster analysis on genus level

**Figure 5:**
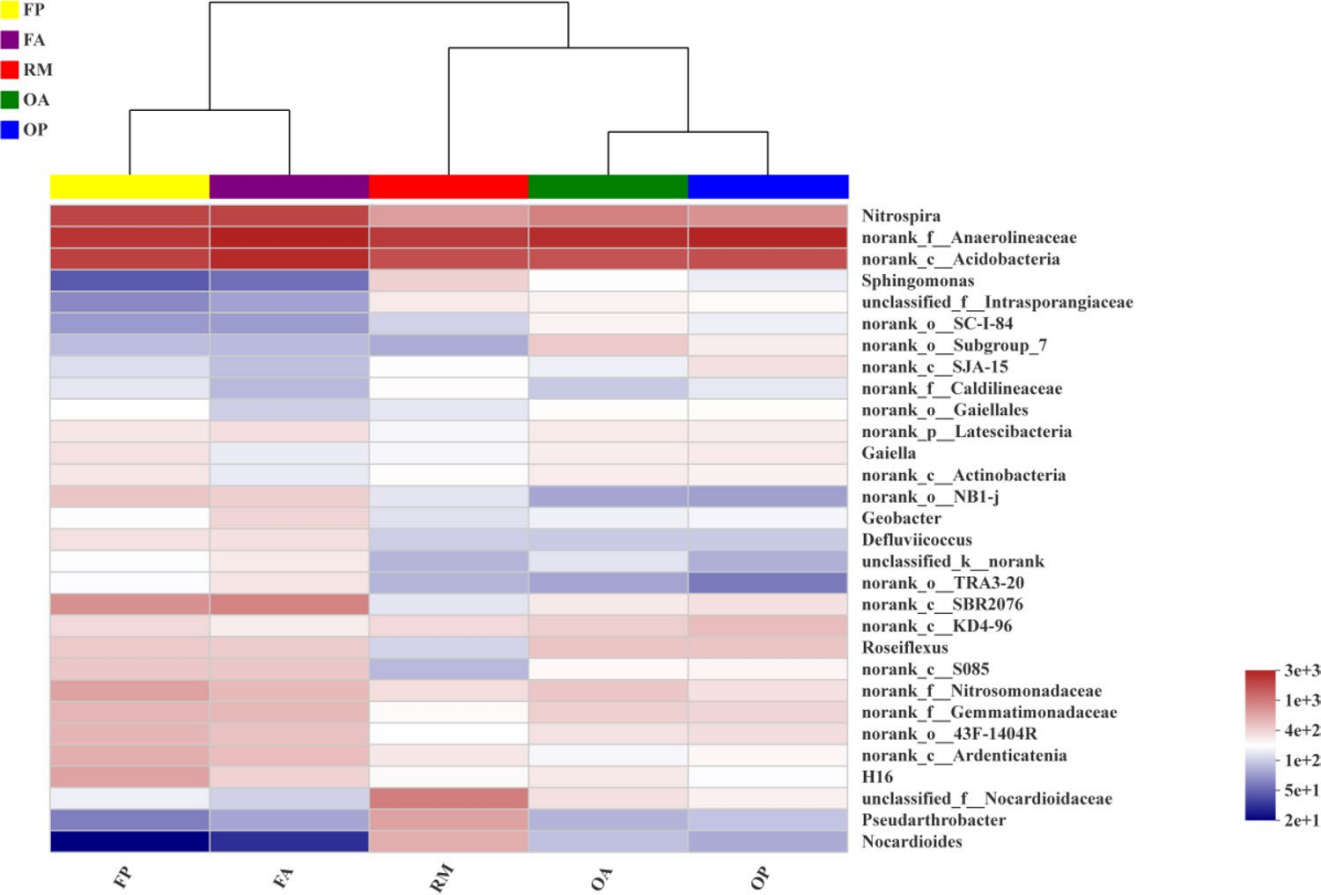
Heatmap of cluster analysis of the top 30 genera in different rice systems and areas.

**Figure 6:**
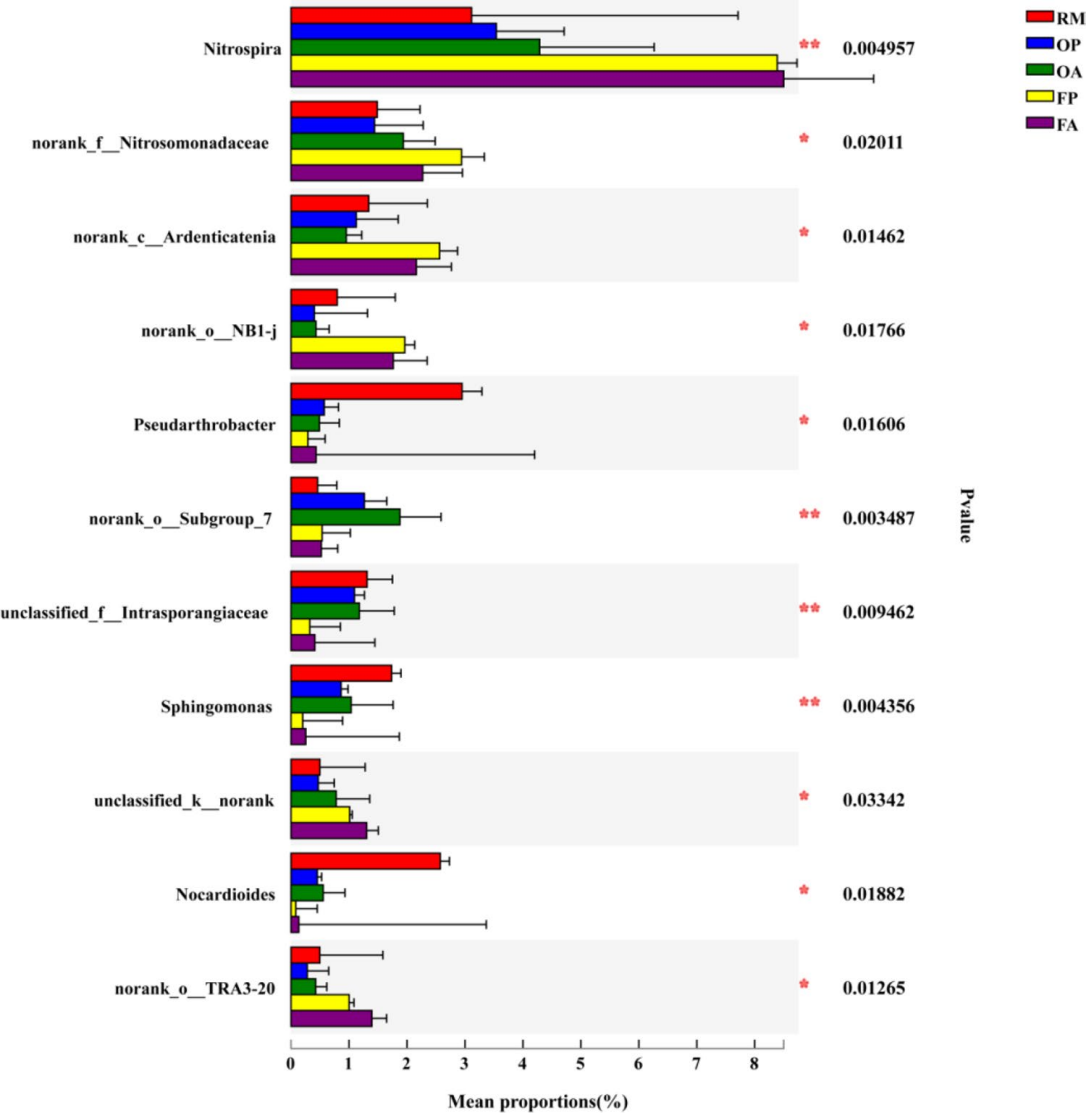
The differences with significance of the top 30 genera in different rice systems and areas (* indicates 0.01 < *P* ≤ 0.05, ** indicates 0.001 < *P* ≤ 0.01).

The community heatmap of the top 30 genera is shown in Fig. 5. The genera *Nitrospira*, *Anaerolineaceae* and *Acidobacteria* showed higher abundances than the other genera. The community composition on genus level also differed markedly across the different experimental groups. The clustering tree indicates that the different areas in a same rice–fish field (i.e. OP vs OA or FP vs FA) showed high similarity on genera composition and clustered together first. Among the different rice farming system, the genera composition was clear distinct with each other. Moreover, the statistical difference among the 5 experimental groups of the top 30 genera was checked with Kruskal–Wallis test. The results showed that 11 genera were observed significant differences among treatments (Fig. 6, only significance phyla presented). Some genera, such as *Nitrospira, norank_f_Nitrosomonadaceae, norank_c_Ardenticatenia* and *norank_o_NB1-j* were enriched in the 5 years of rice–fish field (FP and FA), while some genera, such as *Pseudarthrobacter, Sphingomonas* and *Nocardioides* were enriched in RM. This results indicated that the soil bacterial community composition on genus level has changed greatly since the implementation of rice–fish farming regime, which is consist with previous analysis on phylum level. In addition, we used LEfSe analysis to show the differences in the taxa from the phylum to the genus level among the 5 experimental groups (Figure S1). A total of 150 taxa were observed to have significant differences in abundances, of which 60 taxa were enriched in RM, 27 taxa were enriched in OA, 24 taxa were enriched in FA, 22 taxa were enriched in OP and 17 taxa were enriched in FP.

### Correlation between bacterial community composition and soil properties

**Figure 7:**
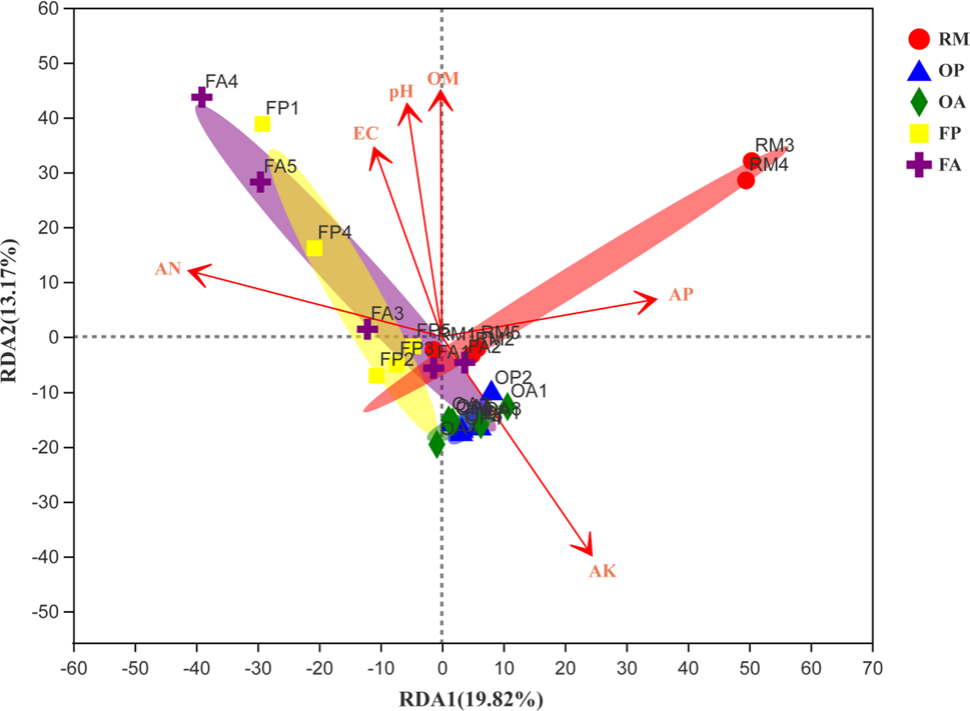
RDA analysis at the OTU level between the soil bacterial communities and soil properties.

Redundancy analysis (RDA) at the OTU level was performed to establish the linkages of soil properties with bacterial community composition (Fig. 7). The results showed that the soil properties together explained 32.99% of the total variations in bacterial community composition. The bacterial community in F_group (FP and FA) was positively correlated with soil factors, including AN and OM content, EC and pH value. In contrast, the bacterial community in O_group (OP and OA) was only positively correlated with soil AK content. In addition, the Mantel test was employed to confirm the significance between soil factors and bacterial community composition. The results (Table 3) indicated that the soil community composition was significantly (*P* < 0.05) correlated with the selected soil factors, except for soil AP content. Soil AK content was the most influential factor that correlated with bacterial community composition.

**Table 3:** The correlations (r) and significance (*P* value) determined by Mantel test between the bacterial community composition and soil factors.

Soil factors	r statistic	*P* value
AN	0.2872	0.014
AP	0.1052	0.276
AK	0.5510	0.001
OM	0.2379	0.020
pH	0.3142	0.002
EC	0.1964	0.024

### Rice yield, quality and economic benefit

Rice yield, several quality indicators and the net economic benefit for the different rice cultivation regimes were also evaluated. As shown in Table 4, rice yield was decreased in rice–fish integrated farming systems, especially in the 1st year of rice–fish field. However, the net economic benefit in rice–fish field of the 5th year was increased due to the high economic value of aquatic animals. In the 1st year of rice–fish farming regime, the aquatic animal was not captured for sale as it had not yet reached the marketable size. Therefore, the net benefit of the 1st year of rice–fish field was lower than that in the rice monoculture. For the quality indicators, the protein content and milled rice ratio of rice–fish field were higher than rice monoculture, while the amylose content was opposite. More details for the quality and benefit analysis of rice–fish integrated farming system could be found in previous publication^5^.

**Table 4:** The rice yield, quality indices and net economic benefit of different rice farming systems.

Indicators	Rice monoculture	Rice–fish field in the 1st year	Rice–fish field in the 5th year
Rice yield (t·ha^−1^)	7.99 ± 0.23	6.91 ± 0.03	7.45 ± 0.29
Protein content (%)	6.86 ± 0.04	7.07 ± 0.07	6.99 ± 0.02
Amylose content (%)	8.50 ± 0.01	8.30 ± 0.06	8.34 ± 0.04
Milled rice ratio (%)	69.30 ± 0.27	75.90 ± 0.25	78.17 ± 0.22
Net economic benefit (1000 RMB·ha^−1^·year^−1^)	20.51 ± 0.71	18.10 ± 0.10	23.23 ± 0.45

## Discussion

Developing Chongming Island into a world-class ecological island is very important for the urban development of Shanghai city. However, one challenge that needs to be overcome to achieve this goal is the high rate of agrochemical applications during the conventional agriculture production on the island. The rice–fish integrated farming regime could address this challenge by providing a means to diversify agricultural and aquacultural production, with increased yields and economic benefits mainly achieved by increased nutrient recycling and decreased agrochemical input^5, 14^. The rice–fish fields generally exhibited improved productivity and enhanced ecological services, which have been reported in many previous studies^15, 16^. However, the fundamental mechanisms for these enhancements in the rice–fish fields are not well-studied, which may limit the further promotion of rice–fish integrated farming regimes.

Soil microorganisms play an important role in regulating soil fertility by changing the diversity and structural composition of soil microbial communities^11, 17^. Our study results with Illumina MiSeq sequencing indicated that soil bacterial community composition in rice–fish field was significantly different with rice monoculture, especially after long-term implementation of rice–fish regime (i.e. 5 years). Five phyla in the top 15 phyla and 11 genera in the top 30 genera were observed with significant differences among treatments. From phylum to the genus level, 150 taxa in total were detected to have significant differences in the abundances. This result indicated that the structure of the soil bacterial community was greatly changed after the rice–fish integrated farming regime was adopted in paddy field. Soil factors, such as AN content may play a crucial role in the differentiation of soil bacterial community composition, which has been supported by RDA analysis. Many researches have demonstrated that the interaction between functional bacteria and soil nutrients supply could enhance the productivity of agricultural systems^18, 19^. However, this study was only provided the basic characteristics of soil bacterial community composition in rice–fish field. The bacterial functions and its relation with soil nutrients supply in the rice–fish field were not studied in depth. The phylum *Nitrospirae*, which observed with the most significance among different rice farming systems, need further studies to explore its functions in soil N transformation in rice–fish field. Previous research has shown that functional bacteria can decompose soil mineral N and improve nutrient availability, thereby promoting nutrient absorption by crops^18^.

Another interesting result in this study is no significant differences of the soil bacterial community composition were observed between the planting area and aquaculture area in rice–fish fields. This finding means that the differentiation of the soil bacterial communities in the rice–fish fields occurred throughout the whole system, not solely in the aquaculture area. This could be attributed to the deeper water and continuous flooding (i.e., no aeration period) in the rice–fish fields, which connected the whole system and provided an interactive environment for the various chemical and biological processes in the soil. This may be another key factor in the high productivity of rice–fish integrated farming system.

## Conclusion

As the implementation of rice–fish integrated farming regime in Chongming Eco-island, the soil properties and bacterial community composition in rice–fish field was significantly different with that in rice monoculture. Significant differences of soil bacterial communities were also observed at both phylum level and genus level among different treatments. Soil properties, such as AN contents, play an important role in the differentiation of soil bacterial composition in rice–fish field. However, this study is only a preliminary exploration on the basic characteristics of soil bacterial community composition in rice–fish integrated farming system and its relationship with soil properties. Further studies are still needed in the direct linkage between soil nutrients supply and the crucial functional microorganisms, thus to reveal the mechanisms of high productivity of the rice–fish integrated system.

## Methods

### Experimental field description

**Figure 8:**
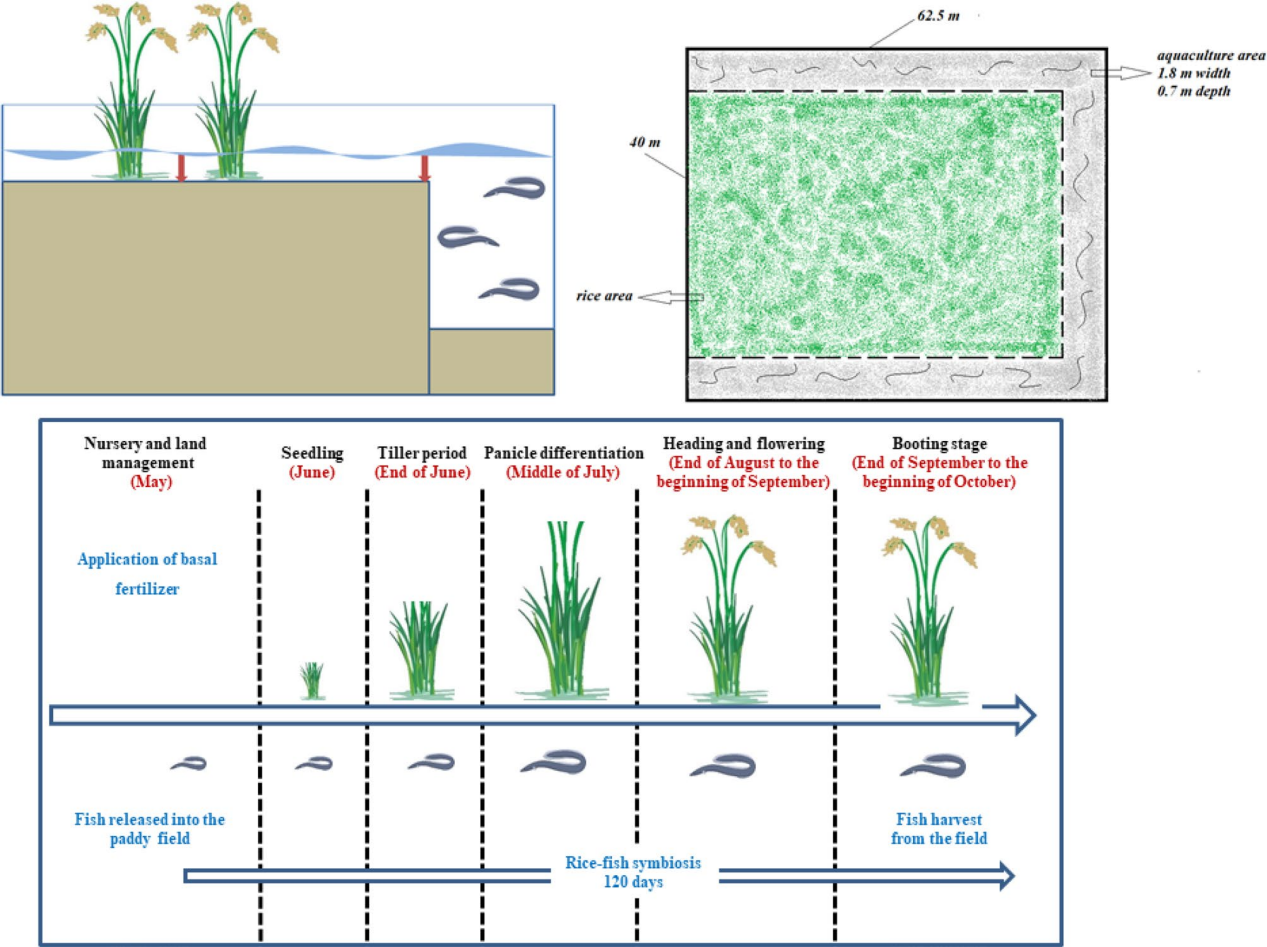
Layout and sequence diagram of the rice–fish integrated farming system (the red arrow indicates the sampling site).

The experimental site is located in Sanxing town, Chongming Eco-island, Shanghai, China (31°46′52″N, 121°15′17″E). This area has a subtropical humid monsoon climate, with a daily average air temperature of 15.6 °C and annual precipitation of 1008 mm. The soil type in this region is classified as Anthrosol based on Chinese Soil Taxonomy. Paddy rice is one of the typical food crops on the island, and the rice cultivation pattern is changing from monoculture to rice–fish integrated farming in recent years. Three paddy fields, including a rice monoculture field and two rice–fish fields with different planting years (1 year and 5 years), were selected to investigate soil bacterial community characteristics. The layout (Fig. 8) of the rice–fish field (40.0 m × 62.5 m) consisted of a rice planting area (38.2 m × 58.9 m) and surrounded on three sides by an aquaculture ditch (0.7 m in depth and 1.8 m in width). The ratio of the rice area to the aquaculture area was 9:1. Yellow finless eel and loach were cultured in the aquaculture area during the rice growing season. Including 2 kg of deep yellow finless eel fry and 2 kg of loach fry were released into the aquaculture area that surrounding the rice fields. In the first year of rice–fish farming system, the finless eel and loach were not captured as they had not reached marketable size. Then, fish will captured from late September to early October before the rice harvest in every rice season (except the first season). The management sequence diagram of the rice–fish regime was shown in Fig. 8. Rice-fallow rotation was performed in the experimental paddy fields following the local conventional agricultural practices. The rice variety of “Qingxiangruangeng” is cultivated in these selected paddy fields. During rice season, each paddy field received 390 kg ha^−1^ of compound fertilizer (15% N, 15% P and 15% K) as the basal fertilizer, and 75 kg ha^−1^ of urea (46% N) was used as topdressing at the seedling, tillering, elongation and booting stages. Flooding irrigation was adopted during the rice season. The rice monoculture field was managed with a midseason aeration period. During this period, the rice field is drained for approximately 10 days, and then re-flooding until the rice is ripe. In contrast, no midseason aeration was conducted in the two rice–fish fields, which carried out continuous flooding for aquaculture.

### Soil sampling and measurements

In August of the 2018 rice season, soil samples were collected from the top-layer (0–20 cm) of the 5 designed treatments, including (1) RM: the rice monoculture field; (2) OP: the planting area in the 1st year of rice–fish field; ((3) OA: the aquaculture area in the 1st year of rice–fish field; (4) FP: the planting area in the 5th year of rice–fish field; (5) FA: the aquaculture area in the 5th year of rice–fish field. The soil sampling sites are indicated in the schematic diagram of the rice–fish integrated farming system (Fig. 8). Five duplicates were collected for each treatment, and 25 soil samples were collected in total. The soil samples were brought back to laboratory immediately in a cold storage box for the extraction of soil microbial DNA. In addition, soil properties, including organic matter (OM), soil pH, available nitrogen (AN), available phosphorus (AP) and available potassium (AK) contents, were also measured with standard experimental methods. The correlation between the soil bacterial communities and soil available nutrients was also analyzed with RDA analysis.

### Soil DNA extraction, PCR amplification and sequencing

Soil microbial DNA was extracted using an E.Z.N.A.® soil DNA Kit (Omega Biotek, Norcross, GA, U.S.). The final DNA concentration and purification were determined by a NanoDrop 2000 UV–Vis spectrophotometer (Thermo Scientific, Wilmington, USA), and DNA quality was checked by 1% agarose gel electrophoresis^20^. The V3-V4 hypervariable regions of the bacterial 16S rRNA gene were amplified with primers 338F (5′-ACTCCTACGGGAGGCAGCAG-3′) and 806R (5′-GGACTACHVGGGTWTCTAAT-3′) by a thermocycler PCR system (GeneAmp 9700, ABI, USA)^21^. The PCRs were conducted as follows: 3 min of denaturation at 95 °C, 27 cycles of 30 s at 95 °C, 30 s for annealing at 55 °C, and 45 s for elongation at 72 °C, and a final extension at 72 °C for 10 min^22^. The resulting PCR products were extracted from a 2% agarose gel and further purified using an AxyPrep DNA Gel Extraction Kit (Axygen Biosciences, Union City, CA, USA). The purified amplicons were pooled in equimolar amounts and paired-end sequenced (2 × 300) on an Illumina MiSeq platform (Illumina, San Diego,USA) according to the standard protocols described by Majorbio Bio-Pharm Technology Co., Ltd. (Shanghai, China). The raw reads were deposited into the NCBI Sequence Read Archive (SRA) database (accession number: PRJNA635212).

### Data processing and analysis

The raw 16S rRNA gene sequencing reads were demultiplexed, quality-filtered by fastp version 0.20.0^23^ and merged by FLASH version 1.2.7^24^ with the following criteria: (i) the 300 bp reads were truncated at any site receiving an average quality score of < 20 over a 50 bp sliding window, and the truncated reads shorter than 50 bp were discarded, reads containing ambiguous characters were also discarded; (ii) only overlapping sequences longer than 10 bp were assembled according to their overlapped sequence. The maximum mismatch ratio of overlap region is 0.2. Reads that could not be assembled were discarded; (iii) Samples were distinguished according to the barcode and primers, and the sequence direction was adjusted, exact barcode matching, 2 nucleotide mismatch in primer matching. Operational taxonomic units (OTUs) with 97% similarity cutoff^25, 26^ were clustered using UPARSE version 7.1^25^, and chimeric sequences were identified and removed. The taxonomy of each OTU representative sequence was analyzed by RDP Classifier version 2.2^27^ against the 16S rRNA database (Silva v138) using confidence threshold of 70%. In addition, ANOSIM test, Kruskal–Wallis test, Wilcoxon tests, Student’s t-test and Mantel test were employed to quantify the statistical differences among treatments.

## Supplementary Information

Supplementary Information.
