# Supplementary material for: Soil bacterial community composition in rice–fish integrated farming systems with different planting years
Source: Sci Rep. 2021 May 25;11:10855. doi: 10.1038/s41598-021-90370-9 (PMC8149657; doi:10.1038/s41598-021-90370-9)
Supplement: Supplementary file 1 — Supplementary Information. [file 41598_2021_90370_MOESM1_ESM.docx]

**Supplementary material for:**

**Title**

Soil bacterial community composition in rice-fish integrated farming systems with different planting years

**Authors**

Zheng Zhao^1^, Changbin Chu^1^, Deping Zhou^1^, Qingfeng Wang^1^, Shuhang Wu^1*^, Xianqing Zheng^1^, Ke Song^1^, Weiguang Lv^1*^

**Affiliations**

1. Eco-environmental Protection Institute of Shanghai Academy of Agricultural Sciences, Shanghai 201403, China

**Corresponding author**

* Shuhang Wu

E-mail address: [wushuhang88@foxmail.com](mailto:wushuhang88@foxmail.com)

* Weiguang Lv

E-mail address: [lvweiguang@saas.sh.cn](mailto:lvweiguang@saas.sh.cn)

Tel.: +86 21 62202446; Fax: +86 21 62202446

Postal address: Eco-environmental Protection Institute of Shanghai Academy of Agricultural Sciences, 1000 Jinqi Rd., Shanghai 201403, P. R. China


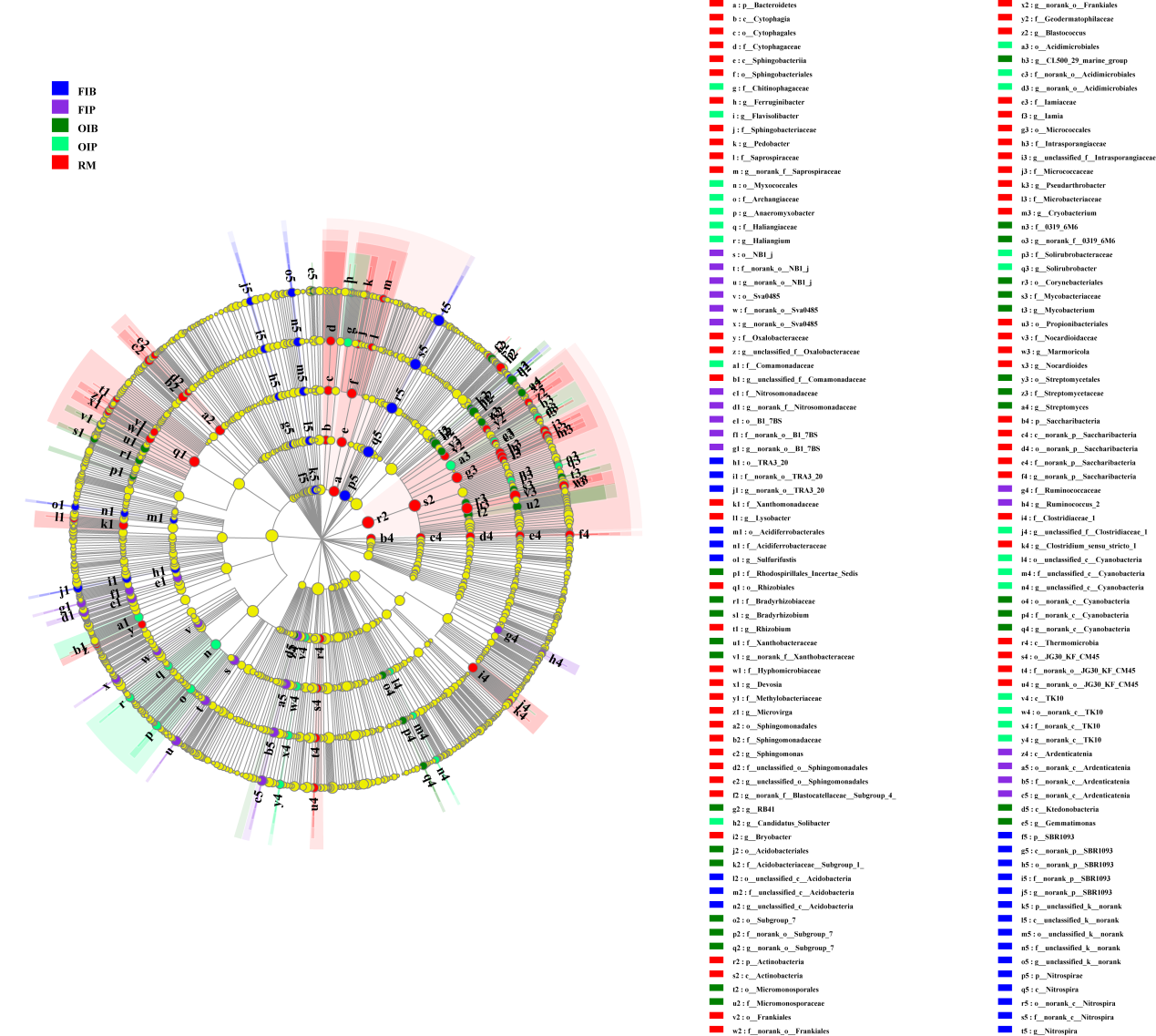


Figure S1 LEfSe analysis of soil bacterial communities from phylum to genus level with LDA scores higher than 3.0 and conducted with all-against-all strict comparative strategy

(Cladograms indicate the phylogenetic distribution of microbial lineages associated with the 5 experimental groups. Each circle's diameter is proportional to the taxon's abundance, and circles represent phylogenetic levels from phylum to genus inside out. Significant differences are represented in the corresponding color of the most abundant taxa. Blue indicates FIB, purple indicates FIP, green indicates OIB, cyan indicates OIP, red indicates RM and yellow indicates no significant difference)
